# Steady-State Visual Evoked Potential-Based Brain–Computer Interface System for Enhanced Human Activity Monitoring and Assessment

**DOI:** 10.3390/s24217084

**Published:** 2024-11-03

**Authors:** Yuankun Chen, Xiyu Shi, Varuna De Silva, Safak Dogan

**Affiliations:** Institute for Digital Technologies, Loughborough University London, London E20 3BS, UK; x.shi@lboro.ac.uk (X.S.); v.d.de-silva@lboro.ac.uk (V.D.S.); s.dogan@lboro.ac.uk (S.D.)

**Keywords:** SSVEP, BCI, EEG, brainwave activity, brain pattern, human activity monitoring

## Abstract

Advances in brain–computer interfaces (BCIs) have enabled direct and functional connections between human brains and computing systems. Recent developments in artificial intelligence have also significantly improved the ability to detect brain activity patterns. In particular, using steady-state visual evoked potentials (SSVEPs) in BCIs has enabled noticeable advances in human activity monitoring and identification. However, the lack of publicly available electroencephalogram (EEG) datasets has limited the development of SSVEP-based BCI systems (SSVEP-BCIs) for human activity monitoring and assisted living. This study aims to provide an open-access multicategory EEG dataset created under the SSVEP-BCI paradigm, with participants performing forward, backward, left, and right movements to simulate directional control commands in a virtual environment developed in Unity. The purpose of these actions is to explore how the brain responds to visual stimuli of control commands. An SSVEP-BCI system is proposed to enable hands-free control of a virtual target in the virtual environment allowing participants to maneuver the virtual target using only their brain activity. This work demonstrates the feasibility of using SSVEP-BCIs in human activity monitoring and assessment. The preliminary experiment results indicate the effectiveness of the developed system with high accuracy, successfully classifying 89.88% of brainwave activity.

## 1. Introduction

**Figure 1:**
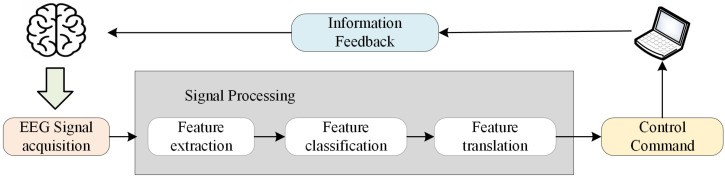
The brain–computer interface framework.

The brain–computer interface (BCI) is a promising technology that can improve the quality for life of people who may have lost the ability to communicate or interact physically with their surroundings using conventional augmentative technologies [1]. It provides a new medium for communication between people and computers by converting brain electrical activity signals into actionable commands to control external devices without the involvement of peripheral nerves and muscles [2]. Many neurophysiological electroencephalogram (EEG) signals have been used to convert human intentions into commands that BCIs can understand, and this EEG signal is called an EEG control signal. Figure 1 illustrates the process flow of BCI technology in converting brain electrical signals into control commands [3].

BCIs can decode neural activity into control commands to trigger wheelchairs [4], prostheses, and many other virtual interface devices. Among the methods of measuring EEG signals, surface EEG is a standard noninvasive technology [5]. Compared with invasive BCI, noninvasive BCI records brain activity by placing EEG signal sensors on the scalp, which can avoid the damage caused by and risks due to surgery and is more applicable and portable in practice [6]. In BCI applications, EEG is a widely adopted noninvasive modality signal, often used in neural instrumentation and measurement (I&M) [7].

Different paradigms are used to establish communication between users and devices. The most widely adopted BCI paradigms include P300 [8], steady-state visual evoked potential (SSVEP) [8], and motor imagery [9]. Among these, SSVEP is a particularly effective and noninvasive method, which detects stable neural responses from the parietal and occipital regions of the scalp induced by periodic visual stimulation [10]. SSVEP is characterized by its ability to elicit a consistent frequency response in an EEG signal corresponding to the frequency of a visual stimulus. This makes it highly suitable for various BCI applications where the user is required to focus on a specific visual stimulus to communicate a command.

SSVEP-BCIs are commonly used in communication applications such as spelling devices, where users select letters or words by focusing on flickering buttons on a screen [11]. SSVEP has been applied in healthcare to develop assistive technologies for individuals with motor disabilities, allowing them to control external devices like wheelchairs or prosthetics through visual focus alone [12]. Additionally, SSVEP is utilized in gaming and virtual reality environments, where users can control interfaces or navigate through virtual spaces with their brain activity.

One of SSVEP’s significant advantages is its high information transfer rate (ITR), which makes it faster than other BCI paradigms. This high ITR is attributed to the direct correlation between the flicker frequency and the neural response, allowing for rapid and accurate detection. Furthermore, SSVEP-BCIs typically require minimal training, as the method relies on natural, involuntary neural responses to visual stimuli. This ease of use and low training burden make SSVEP a practical choice for many users, including those without prior BCI experience [13,14].

However, the current BCI systems suffer from a few limitations that have impeded the development and applications of BCI in real-life scenarios. These limitations include a small number of BCI control commands, resulting in difficulty with the precise control of argumentative devices; the greatly varying accuracy of brain activity recognition among individual participants, rendering the BCIs unreliable; and a slow recognition speed, rendering it unable to meet the requirements of practical applications in real life [15].

The existing public datasets in the field of SSVEP-BCIs mainly focus on spelling tasks or simple command selection, where the data were collected from participants’ reactions to stimuli flashing on a screen. These datasets lack the immersive and dynamic environment required to effectively evaluate BCIs in real-world applications [16]. In contrast, our dataset comprises data generated from a VR-based driving task, providing participants with an immersive and multidirectional control experience that better represents real-world use cases, such as in an assistive technology. This setting offers richer and highly dynamic brain activity data compared to the existing datasets, making it suitable for exploring more complex, continuous BCI control scenarios [17].

In this paper, we provide an SSVEP-BCI system with multifrequency stimulation for a virtual driving environment, where a brain-controlled vehicle (BCV) obtains control commands through a BCI analyzing the driver’s EEG signals. To develop an effective SSVEP-BCI system, we also propose a new shared control method [18] that integrates brain control with fuzzy logic [19], enabling the system to account for and interpret the user’s subjective intentions more accurately. Since fuzzy control does not require a specific mathematical model, it avoids the thorny problem of modeling the decision-making process [19]. Fuzzy discrete event system (FDES) supervision theory, which is widely used in mobile robot control, is also introduced to supervise the subject’s control commands to increase command recognition accuracy [20,21]. Based on the accuracy of the participant’s command, the decision result of the automatic fuzzy controller is then adjusted to better match the participant’s intention. We created multiple visual stimuli corresponding to forward, backward, left, and right movements as the basis for brain-controlled cars. We also completed a set of experiments to collect EEG data to build an SSVEP-BCI dataset.

The rest of this paper is organized as follows: Section 2 reviews the SSVEP-BCI-related work in the literature, providing a comprehensive understanding of the existing knowledge. Section 3 introduces the research methods used in this study, ensuring the validity and reliability of the results. Section 4 describes the experiment design, detailing the major aspects of the experimental process applied. Section 5 evaluates the proposed system’s performance through a preliminary trial, highlighting its effectiveness. Section 6 discusses the results. Section 7 concludes this paper.

## 2. Related Work

SSVEP-BCIs are critical in practical applications, particularly smart health and medical assistance. For individuals with severe physical disabilities, such as amyotrophic lateral sclerosis (ALS) [22] or locked-in syndrome [23], BCI technologies offer a means to communicate and interact with their environment, which would otherwise be impossible. SSVEP-BCI provides these individuals with a noninvasive and user-friendly solution, enabling them to control communication devices, computer systems, and home automation systems using their thoughts [24]. This ability to engage with their surroundings significantly enhances their independence and quality of life [25]. For example, patients with locked-in syndrome, who retain full cognitive function but cannot move any part of their body except for limited eye movements, can benefit from SSVEP-BCI systems integrated with eye-tracking technology. This combination allows them to interact with their environment by focusing on specific visual stimuli on a screen, effectively enabling communication and control over external devices [26]. This approach not only accommodates the limited physical capabilities of these patients but also provides a seamless and reliable means of interaction, offering a significant improvement in their quality of life. Cao et al. [27] proposed a high-speed online speller based on SSVEP-BCI to help patients with locked-in syndrome express themselves to others. SSVEP-BCI devices can also help patients with motor neuron disease (MND) control wheelchairs through EEG signals [4]. In addition, Shyu et al. [28] developed an SSVEP-BCI command platform that enables patients with paralysis to perform operations, such as adjusting volume, changing channels, or selecting movies, on multimedia devices. To achieve quadcopter control, Wang et al. [29] designed a mobile BCI system using the SSVEP paradigm, which allows users to accurately and smoothly complete 3D flight instructions. SSVEP-BCI devices can partially restore the user’s ability to communicate with the physical environment and greatly improve the quality of life of users who have lost mobility or language ability. Healthy users can also use these devices to control external devices to enhance the diversity of and possibilities in life.

Despite the great potential of the SSVEP-BCI, its development and deployment are hampered by a significant challenge: the limited availability of comprehensive EEG datasets. While research on multifrequency SSVEP-BCIs has been conducted on the limited available datasets, including those by Shyu et al. [30] and Asheri et al. [31] with three visual stimuli of different frequencies, the field still lacks extensive datasets that explore more complex frequency interactions and applications beyond basic paradigms. Since the release of the SSVEP-BCI benchmark dataset in 2017 [32], which has been cited in over 200 academic works, progress has been made, particularly in developing datasets for BCI spellers [33]. However, most existing datasets focus on single-frequency SSVEP [34], and there remains a need for publicly available datasets specifically designed for SSVEP-BCI applications involving multifrequency paradigms with moving objects as stimuli, which are crucial for advancing research in SSVEP-based motion control and interaction.

Lim and Ku [35] proposed a multicommand single-frequency SSVEP-BCI system using flickering action videos. In the work, the participants activated visual stimuli based on video content, targeting left, right, and rest actions. The participants identified these actions by watching a flickering action video accompanied by left and right elbow movements. The system achieved a classification accuracy of 74.60% using the common spatial pattern (CSP) algorithm.

Asheri et al. [31] introduced open-source OpenVibe software to test SSVEP. They used a public SSVEP dataset containing three visual stimuli with 12, 15, and 20 Hz stimulation frequencies. Using the CSP algorithm, the classifier’s highest accuracy was 85.10%, 84.56%, and 86.83% with linear discriminant analysis (LDA) and 87.92%, 89.62%, and 90.57% with support vector machine (SVM) for these respective frequencies.

Many studies have shown that combining SSVEP-BCI systems with VR environments can provide an enhanced, immersive user experience beneficial for neurorehabilitation, educational tools, and gaming applications. Wen et al. [36] demonstrated that SSVEP-based BCI systems could be effectively integrated with VR to improve user engagement and motivation in neurorehabilitation tasks. Similarly, Zehra et al. [37] discussed the unique benefits and challenges of using SSVEP-BCI systems in VR, including the need for realistic and responsive environments to achieve high-quality user interaction. These studies highlight the potential of combining BCI with VR to create immersive and practical applications that can significantly enhance user experiences.

In this research, we conducted a preliminary experiment and analyzed the experimental results of one male healthy participant aged 26 with a university degree. In our future work, we will create an EEG dataset with 20 healthy participants based on the multifrequency SSVEP-BCI paradigm. With forward, backward, left, and right movement as visual stimuli, the dataset includes EEG recordings captured using a 16-channel EEG acquisition system. Open access to high-quality EEG data is essential for researchers to develop, test, and evaluate new BCI algorithms and applications. This data collection effort aimed to provide the scientific community with a valuable resource to advance the development of SSVEP-BCIs.

Integrating BCI with virtual environments offers exciting possibilities for creating immersive and interactive applications. In this study, we developed a virtual environment [38] to enable the control of a virtual vehicle with an SSVEP-BCI system. This setup demonstrates the practical application of SSVEP-BCI in controlling objects and highlights the potential for such systems to be used in various human activity settings. For example, people with limited mobility could use similar systems for assisted driving or navigation in virtual environments for therapeutic purposes.

## 3. Research Methodology

This study presents an enhanced methodological approach for interpreting EEG signals in BCI applications, particularly within Unity-based virtual environments [39]. The CSP algorithm is used for feature extraction to classify the collected multichannel EEG data. The independent component analysis (ICA) method decomposes multiple mixed signals into independent additive components [40].

### 3.1. Common Spatial Pattern Algorithm

CSP is a type of spatial filtering algorithm commonly used in BCI classification. It generates features for classification using spatial filters to enhance the variance discrepancies between two signal classes [41,42].

Let $X_{i}\in R^{\left(C\times S\right)},i=1,2,\ldots ,N$ represent the collected EEG signals of *N* samples with two imaginary tasks $\left\{V_{+},V_{-}\right\}$, where *C* is the number of EEG channels, and *S* is the total number of sampling points. The goal of the CSP algorithm is to find a mapping matrix $W\in R^{\left(C\times C\right)}$ that projects the original EEG signals to a new space $X_{csp}=WX$, with each row vector $w_{*}^{T}$ of matrix $W=(w_{1}^{T},w_{2}^{T},\ldots ,w_{C}^{T})$ being a spatial filter, with *T* representing the transpose operation.

CSP assumes signals from different EEG channels are independent. Therefore, the spatial covariance matrix in the new space, $\Sigma_{csp}=X_{csp}X_{csp}^{T}=\left(WX\right){\left(WX\right)}^{T}=W\left(XX^{T}\right)W^{T}$$=W\Sigma W^{T}$, should be diagonal, i.e., $W\sum W^{T}=\Lambda$, with Σ being the covariance matrix estimated as

(1) $$\Sigma =\frac{1}{N}\sum_{i=1}^{N}\frac{X_{i}X_{i}^{T}}{trace(X_{i}X_{i}^{T})},$$

where $trace(X_{i}X_{i}^{T})$ is the sum of the diagonal elements of matrix $X_{i}X_{i}^{T}$. The trace function is used to normalize the covariance matrices.

To enhance the discriminability between the two classes of EEG signals, the covariance matrices $\Sigma_{+}$ and $\Sigma_{-}$ of the two task classes, $V_{+}$ and $V_{-}$, and their sum Σ, are also calculated as follows:

(2) $$\begin{array}{cc} \Sigma_{+} & =X_{+}X_{+}^{T},\\ \Sigma_{-} & =X_{-}X_{-}^{T},\\ \Sigma & =\Sigma_{+}+\Sigma_{-}, \end{array}$$

where $X_{+}$ and $X_{-}$ represent the EEG data matrices for classes $V_{+}$ and $V_{-}$, respectively. The mapping matrix W should satisfy the following conditions:

(3) $$\begin{array}{cc} W\Sigma_{+}W^{T} & =\Lambda_{+},\\ W\Sigma_{-}W^{T} & =\Lambda_{-},\\ W\Sigma W^{T}=W\left(\Sigma_{+}+\Sigma_{-}\right)W^{T} & =\Lambda_{+}+\Lambda_{-}=I, \end{array}$$

where $\Lambda_{+}$ and $\Lambda_{-}$ are diagonal matrices, and *I* is the identity matrix.

Matrix W can be simply found by solving the generalized eigenvalue problem:

(4) $$\Sigma_{+}w=\lambda \Sigma_{-}w,$$

where $w\in \{w_{1},w_{2},\ldots ,w_{C}\}$ is the generalized eigenvector (as a column vector), and $\lambda \in \{\lambda_{1},\lambda_{2},\ldots ,\lambda_{C}\}$ is the corresponding eigenvalue.

As W is composed of generalized eigenvectors $w_{j}$ (j=1,2,…,C) of (4), $\lambda_{j}^{+}=w_{j}^{T}\Sigma_{+}w_{j}$ and $\lambda_{j}^{-}=w_{j}^{T}\Sigma_{-}w_{j}$ are the corresponding diagonal elements of $\Lambda_{+}$ and $\Lambda_{-}$; when $\lambda =\lambda_{j}^{+}/\lambda_{j}^{-}$ the conditions in (3) are satisfied [43].

The generalized eigenvalue, λ, indicates the discriminability between the two task classes. It is obvious that to obtain more discriminative features for classification, the difference between $\lambda_{j}^{+}$ and $\lambda_{j}^{-}$ should be as large as possible. After obtaining the mapping matrix W, the eigenvectors corresponding to the *m* largest and *m* smallest eigenvalues are selected as the spatial filters, with *m* being the number of components selected for feature extraction. Finally, the feature vector for classification can be calculated as

(5) $$f_{p}=\log\left(\frac{\mathrm{Var}(X_{csp}\left(p\right))}{\sum_{i=1}^{2m}\mathrm{Var}\left(X_{csp}\left(i\right)\right)}\right),\;p=1,2,\ldots ,2m,$$

where Var(·) denotes variance, and the logarithm operation log(·) here is used to approximate a normal distribution of the feature data [44].

### 3.2. Independent Component Analysis

ICA is a powerful technique for separating multivariate signals into their underlying independent sources [45]. ICA uses the central limit theorem, which states that the sum of independent random variables tends to be more Gaussian than the individual variables to identify independent components through estimating their non-Gaussianity [46]. As a blind source separation method, ICA is often used in applications where mixed signals must be separated without prior information about the mixing process, such as separating different sound sources in a room or isolating EEG signals corresponding to different brain activities [47]. In practice, the FastICA algorithm is widely used due to its computational efficiency. It iteratively maximizes the non-Gaussianity to estimate independent components and is faster than other gradient-based methods [48].

Let $x={\left[x_{1},x_{2},\ldots ,x_{n}\right]}^{T}$ be an observed signal vector, where each $x_{i},i\in \left[1,n\right]$ is a linear mixture of *n* unknown independent components $s_{j},j\in \left[1,n\right]$. The ICA model can be represented as

(6) x=As,

where A is an unknown mixing matrix and $s={\left[s_{1},s_{2},\ldots ,s_{n}\right]}^{T}$ is a vector of statistically independent components.

The objective of ICA is to estimate both the independent components s and the mixing matrix A using the observed signals x. This can be achieved by finding a demixing matrix W such that

(7) s=Wx.

One approach to estimating W is to maximize the non-Gaussianity of the components of s. This can be achieved by optimizing the negentropy of each component. The negentropy of s is defined as

(8) $$J\left(s\right)=\sum_{i=1}^{n}\left[H\left(s_{i_{\mathrm{gaussian}}}\right)-H\left(s_{i}\right)\right],$$

where $H(s_{i})$ is the entropy of component $s_{i}$, and $H(s_{i_{\mathrm{gaussian}}})$ is the entropy of a Gaussian random variable with the same variance as $s_{i}$.

The FastICA algorithm finds the maximum of the negentropy through multiple iterations of demixing W. In each iteration step *k*, the weight vector u is updated as

(9) $$u^{\left(k+1\right)}=E\left[xg(u^{(k)T}x)\right]-E\left[g^{\prime }\left(u^{(k)T}x\right)\right]u^{\left(k\right)},$$

where E denotes the expected value, *g* is a nonlinear function, and $g^{\prime }$ is its derivative. After each update, u is normalized.

## 4. Experimental Setup

### 4.1. Participants

As a preliminary trial to examine the system setup and its effectiveness, data were collected from one participant. In the next data collection phase, we plan to collect EEG data from 20 healthy participants (aged between 18 and 30 years old) with normal or corrected normal vision strength and no visual epilepsy. All participants will be first-time SSVEP-BCI users. Before the experiment, the participants will be asked to familiarize themselves with the experimental protocol and informed of their rights to withdraw from the experiment at any time. At the beginning of the experiment, they will be asked to read and sign an informed consent form prepared for this study. The necessary research ethics approval was obtained for this study.

### 4.2. EEG Setup

**Figure 2:**
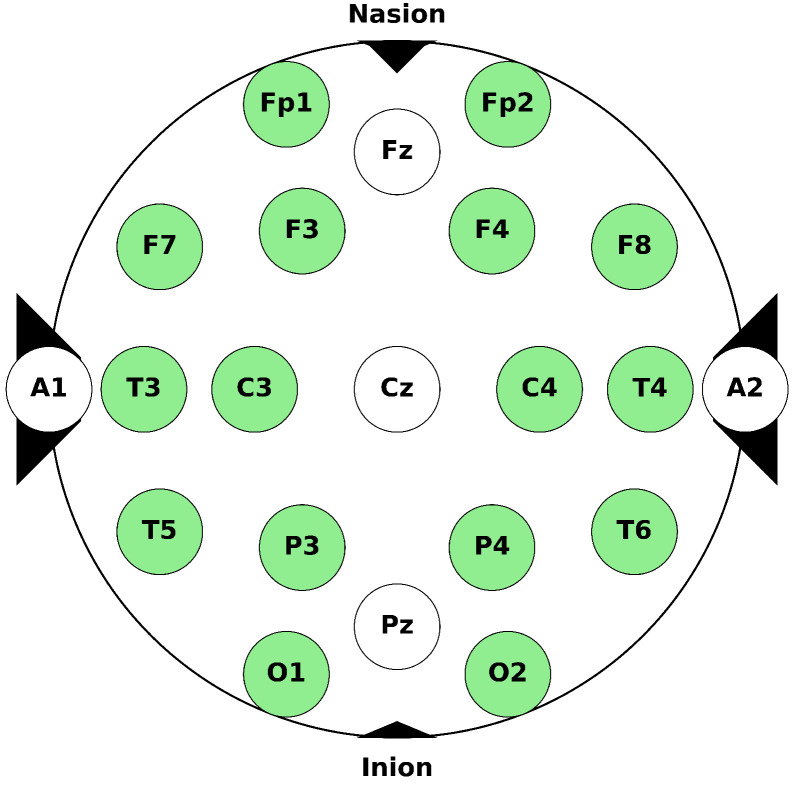
The locations of electrodes in an International 10–20 system for EEG recording. The 16 electrodes marked with colors represent the 16 channels used in this research experiment.

The EEG signals were recorded in a room without any electronic equipment interference using an OpenBCI device [49]. According to the International 10–20 system [50], the electrode impedance was kept below 10 kΩ. As shown in Figure 2, brain activity was initially recorded from 16 channels, specifically Fp1, Fp2, F3, F4, C3, C4, P3, P4, F7, F8, T3, T4, T5, T6, O1, and O2. Among them, the eight most effective channels, F3, F4, C3, C4, P3, P4, O1, and O2, were chosen for data analysis and classification based on the significance of their contribution to SSVEP-BCI performance. This was expected due to their positioning corresponding to the motor and visual cortical areas, as supported by previous studies [51]. In turn, this ensured that the most relevant signals were analyzed, optimizing the classification performance and reducing computational complexity. Additionally, Figure 2 includes electrodes Fz, Cz, Pz, A1, and A2 for reference purposes. Though not used in the current study, these electrodes are part of the full 10–20 system layout, with A1 and A2 serving as reference electrodes and Fz, Cz, and Pz providing standard midline placements.

A1 (left mastoid) and A2 (right mastoid) electrodes serve as the reference and ground electrodes, respectively. The OpenBCI device uses conductive gel through wet electrodes to achieve low impedance, high signal quality, and long-term stability.

During the data acquisition process, the EEG data and synchronous firing signals were recorded at a sampling rate of 250 Hz and saved to data files for offline analysis. In the OpenBCI device setup, a built-in 50 Hz notch filter was used to remove power line noise, and a bandpass filter (BPF) was set from 0.1 Hz to 100 Hz to preserve the broadband spectral characteristics.

### 4.3. Experimental Procedure

**Figure 3:**
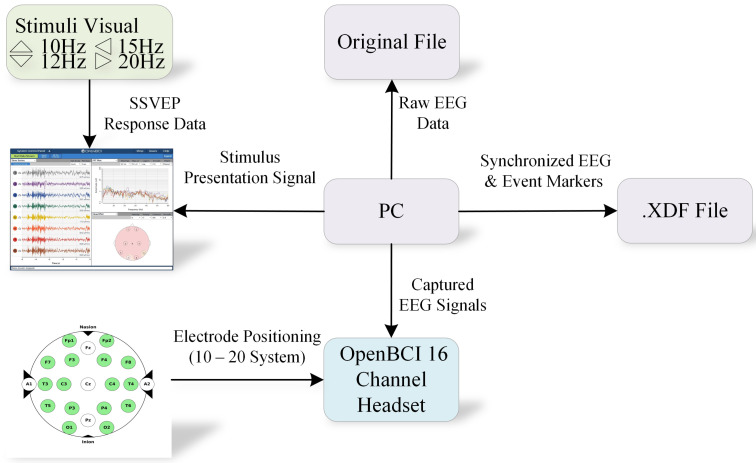
Experimental setup: The computer (PC) is outside the acquisition room and runs the stimulation protocol. The OpenBCI device records the participant’s EEG signals based on the electrode distribution of the International 10–20 system. The PC then receives the recorded EEG data from the acquisition system and records all the present event information. An .xdf file is created and saved at the end of the recording. At the same time, the original EEG signal data file is also saved.

It took about an hour for participants to familiarize themselves with the experiment’s configuration, procedure, and environment. During the experiment, the participants were seated in a comfortable chair in front of a computer display with a 60 Hz refresh rate, positioned 60 cm away, which presented the visual cues. Event markers were transmitted using the lab streaming layer (LSL) [52] to ensure precise synchronization between EEG data acquisition and stimulus presentation. LSL is an open-source framework that collects time-synchronized measurements from various devices and applications. All participants were instructed to focus their attention on the center of the display and minimize eye movements and blinks during stimulus presentation. Figure 3 illustrates the main experimental setup.

The display used in the experiment was a 28-inch LCD monitor with a 3840 × 2160-pixel resolution. The visual stimulus protocol was designed using Psychtoolbox [53] running in MATLAB [54], presenting four visual stimuli corresponding to the forward, backward, left, and right directions. The stimuli were arrows displayed on a black background at frequencies of 10, 12, 15, and 20 Hz for forward, backward, left, and right directions, respectively. These frequencies were chosen to ensure minimal overlap between the stimulation frequencies, thereby enhancing the discrimination between the corresponding EEG responses. Specifically, the frequencies were selected to avoid the interference of their harmonics and subharmonics, because interference would complicate the interpretation of the SSVEPs due to the potential overlapping responses in the EEG signal [55]. Additionally, the chosen frequencies spanned a range that was shown to elicit robust SSVEP responses in previous studies [13] while minimizing participants’ fatigue and discomfort. Each stimulus was preceded by a 1 s prompt, during which a smaller arrow was displayed to indicate the direction of the upcoming stimulus. Each stimulus was randomly presented to ensure the randomness of the experiment. Furthermore, the number of times each frequency representing the four directions was presented was balanced across the experiment. The experiment consisted of 100 SSVEP trials and was divided into two groups of 50 trials each. Each trial lasted 12 s, including 1s prompt, 5 s of flickering stimulus, 4 s of blank screen, and 2 s of rest. An additional 1 min rest period was provided after the first 50 trials.

The sinusoidal flickering method uses Psychophysics Toolbox Version 3 (PTB-3) under MATLAB R2023b. The grayscale value s(*f*, *i*) of the flickering stimulus sequence is calculated as follows:

(10) Low-Depth:Low-Depth:s(f,i)=0.149sin2πfifr+π2+0.464,

(11) High-Depth:High-Depth:s(f,i)=0.5sin2πfifr+π2+0.5,

where $f_{r}$ represents the refresh rate of the screen (60 Hz), *i* is the frame index of the sequence, and *f* indicates the stimulus frequency.

**Figure 4:**
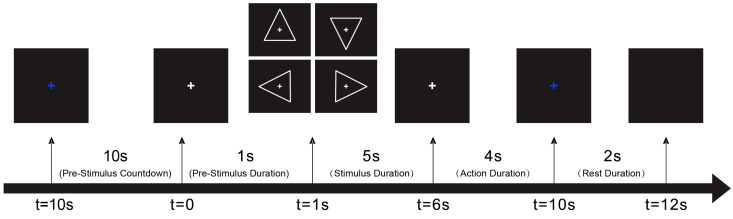
Experimental process in each participant’s experiment.

As shown in Figure 4, a 10 s countdown appeared on the screen at the beginning of the experiment. After that, an arrow appearred for 1 s (pre-stimulus countdown) to hint at the coming stimulus, reminding the participants to concentrate.

Then, the stimulus appeared and flashed for 5 s (stimulus duration) at the frequency depending on its direction. During this period, the participants needed to focus on the stimulus displayed in the center of the screen and avoid blinking. After the flashing stopped and stimulus disappeared, the participants had 4 s (action duration) to recall the previously shown stimulus. The 4 s delay is set based on the latency of the visual system, allowing for the capture of delayed neural activity [32]. Then, there was a 2 s (rest duration) break.

EEG data were collected from the start of the pre-stimulus duration to the end of the rest period, shown as t = 0 and t = 12 s in Figure 4.

Immediately after the experiment, the participants were asked to rate their experience. The participants needed to

- Score each stimulus on a five-point scale based on their level of comfort of the experiment, with 1 to 5 corresponding to very uncomfortable, uncomfortable, slightly uncomfortable, comfortable, and very comfortable, respectively.
- Score each stimulus on a five-point scale based on their perception of the effect of the stimulus flicker, with 1 to 5 corresponding to very annoying, annoying, slightly annoying, noticeable, and imperceptible, respectively.
- Score each stimulus on a five-point scale based on their preference of the stimuli, with 1 to 5 corresponding to very annoying, annoying, neutral, like, and very like, respectively.

### 4.4. Virtual Environment for Assisted Vehicle Maneuvring

**Figure 5:**
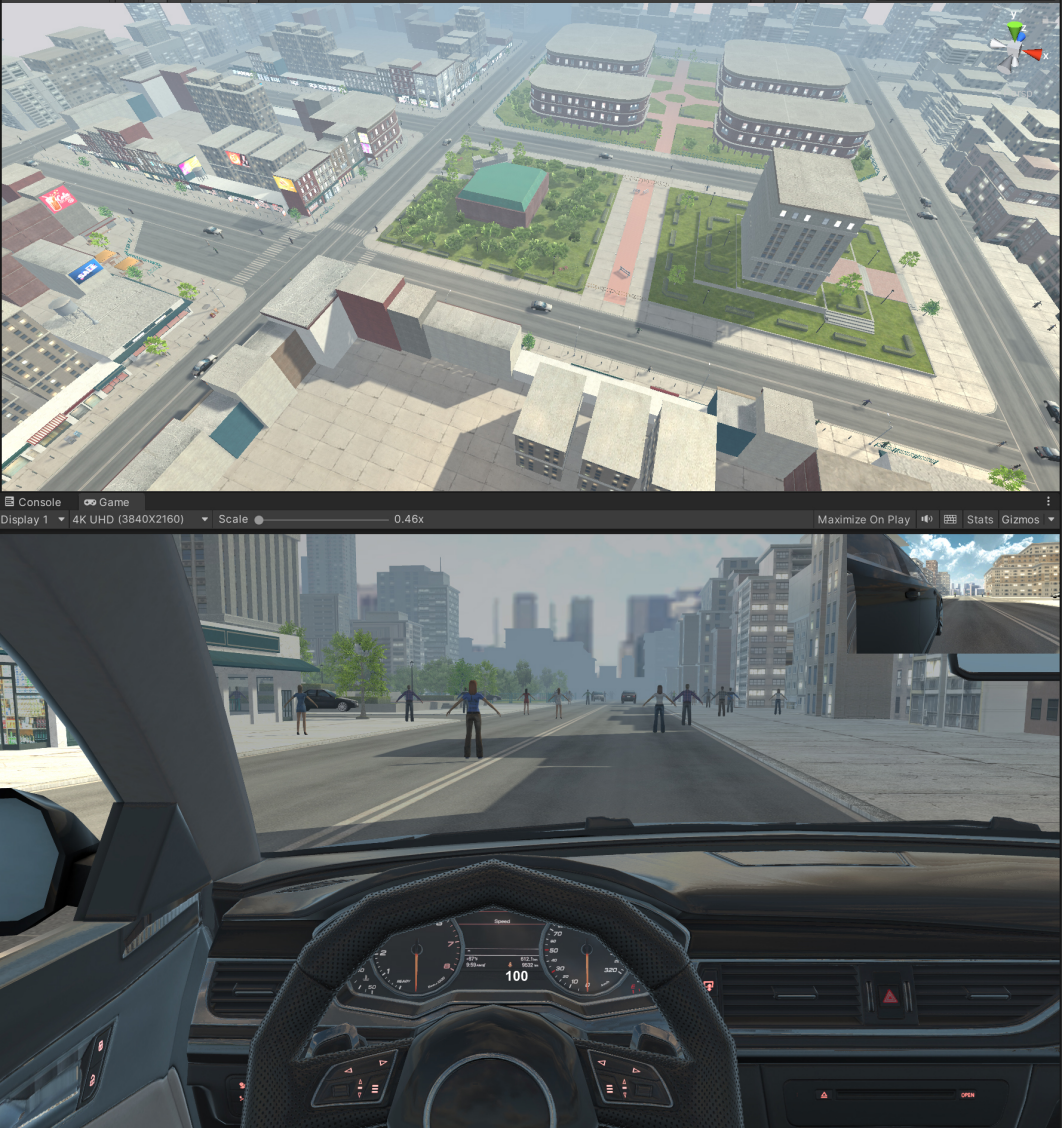
The virtual environment in Unity.

An immersive VR driving environment was created using the Unity platform to enhance the object control ability of the participants through a BCI. The VR setting, shown in Figure 5, featured a city landscape, including buildings, pedestrians, streets, and moving vehicles, simulating real driving conditions. The participant’s task was to operate a virtual vehicle moving forward with acceleration, deceleration, and left and right commands, avoiding collision with objects or pedestrians on the road. The vehicle’s default movement was forward, with acceleration and deceleration to increase and decrease its velocity. The experiment required continuous control of the virtual vehicle through real-time commands. For example, accelerating the vehicle from 20 to 40 m/s required the participant to maintain a constant acceleration command. The virtual driving experiment took six minutes.

The BCI system was connected to the Unity virtual environment using the LSL protocol, which provides a multiplatform data exchange interface between a BCI system and many software applications including Unity. The EEG signals were collected as described in Section 4.2 and Section 4.3. These signals were then processed to extract relevant features, with the participant’s intended action (e.g., accelerate, decelerate, turn left, or turn right) being classified based on the extracted features. The details of data processing and classification algorithms are described in Section 5 and Section 6. These classified actions were further sent to Unity via LSL to control the movements of the virtual vehicle. This setup created a closed-loop system that allowed the participant to directly control the vehicle in a driving scenario, effectively demonstrating the potential of BCI-driven control systems for real-world applications.

## 5. Data Processing

### 5.1. Noise Reduction

The raw EEG data collected in the experiment were filtered through a BPF filter with a bandpass range of 8–50 Hz to mitigate noise and artifacts. The filtered signal was further divided into five distinct frequency bands [56], as follows:

- Alpha waves: 8–12 Hz;
- Beta 1 waves: 12–20 Hz;
- Beta 2 waves: 20–30 Hz;
- Gamma waves: 30–50 Hz.

This division was critical as it allowed us to examine the signal more thoroughly and comprehensively. Alpha waves are the most prominent signal in the EEG data, occurring when the participants are awake. Beta waves are associated with the participants concentrating and processing information, and gamma waves are difficult to detect as their amplitudes are very low [56]. In this work, only alpha and beta waves were used.

### 5.2. Removal of Artifacts with ICA

**Figure 6:**
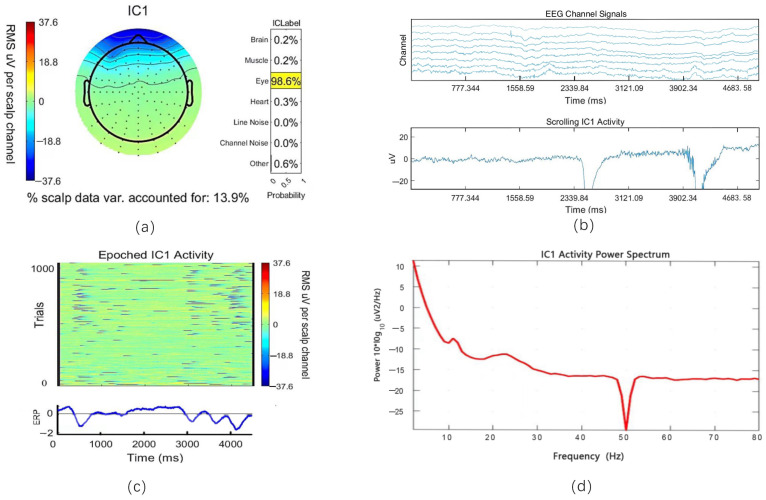
ICA of EEG signal. (**a**) The spatial topography of the first independent component IC1 indicates that 98.6% of its variance is attributable to eye-related artifacts, as highlighted by the ICLabel classification. (**b**) The scrolling activity of IC1 over time shows significant fluctuations, likely due to eye movements or blinks, which are typical sources of artifacts in EEG data. (**c**) Heatmap of IC1 activity with event-related potential (ERP) waveforms summarizing the average activity. (**d**) The power spectrum of IC1 shows significant low-frequency activity and a clear dip at 50 Hz due to the applied notch filter.

The EEG data were subjected to ICA analysis to remove artifacts and improve their quality and interpretability. The data were first centered during preprocessing by subtracting and whitening the mean to standardize the components’ variance. ICA was then applied to decompose the data into statistically independent sources. Figure 6 shows the ICA results of a short period of EEG signal. It reveals that the first independent component, IC1, predominantly represents the eye movement artifacts, accounting for 98.6% of this component’s variance. We can observe the brain activity energy topography of IC1 (Figure 6a), its temporal activity (Figure 6b,c), and its power spectrum (Figure 6d). These visualizations help identify artifacts and guide the process of removing them from the data. This step is crucial as it isolates the neural activity of interest by eliminating components corresponding to non-neural artifacts, resulting in cleaned EEG data suitable for further analysis and classification.

**Figure 7:**
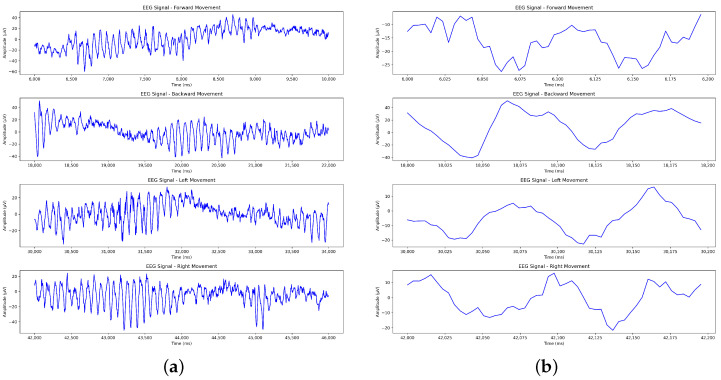
EEG signals from the O1 channel for forward, backward, left, and right movements. (**a**) The EEG signals of each movement during a 4 s action period. (**b**) The EEG signals of each movement during the first 200 ms for viewing details.

Figure 7 depicts the unique neural activities associated with forward, backward, left, and right movements. Figure 7a shows consistent amplitude fluctuations with clear peaks around the middle of signal, which may indicate specific neural responses related to executing the forward action. It also shows that the amplitude of the backward movement initially decreases, followed by more consistent changes, indicating a unique neural adaptation phase before a stable response is achieved. The EEG signals of the left and right movements show rhythmic oscillations in the second half of the signal, suggesting possible activation of movement-related cortical areas involved in lateral movement control. Figure 7b presents the waveforms of the first 200 ms for the four movements at a finer scale, showing more details regarding the signal variation over time. The forward movement showed a relatively smooth decrease in amplitude followed by mild oscillations. The backward movement showed a distinct peak, possibly related to the initial force or intention of the movement. The left movement waveform had moderate fluctuations, indicating activation periods that may be related to lateral movement planning. The right movement showed a mixture of oscillatory patterns, initially decreasing and increasing, reflecting a more variable neural response. These short segments demonstrate that despite similarities in the overall EEG patterns of the movements, their temporal evolution and pattern are quite different, which can be used for classification purposes. This detailed analysis provides insights into how the motor cortex is activated differently in various movement directions, reflecting the specificity of neural control mechanisms during motor tasks.

### 5.3. Feature Extraction with CSP

**Figure 8:**
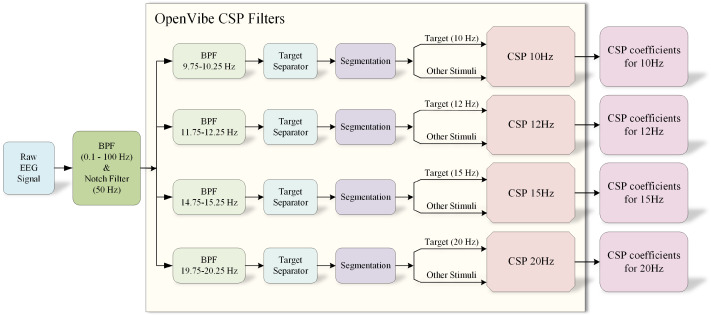
The OpenVibe CSP Filter used to calculate CSP coefficients for the four stimuli at 10, 12, 15, and 20 Hz.

We systematically analyzed the frequency band power corresponding to the four SSVEP stimulation frequencies (10, 12, 15, and 20 Hz) to extract features from the EEG signals. Figure 8 shows that the raw EEG signals are initially processed through the OpenVibe CSP Filters. These filters help isolate the frequency components corresponding to the SSVEP stimuli. The Target Separator, implemented via ICA, separates the independent components for each frequency band.

The data undergo additional processing steps in the OpenVibe CSP Filter (version 2.2.0), including data segmentation and CSP analysis. In the segmentation, a moving window of width 0.5 s in time is used, with each window containing the data of a segment. The window moves forward 0.1 s for the next segment, and this process is repeated throughout the dataset. The segmentation ensures the generation of robust feature vectors by the CSP algorithm. The CSP coefficients from the CSP analysis are taken as the extracted features for further classification.

## 6. Results and Discussion

### 6.1. Classification of Brain Activity

**Figure 9:**
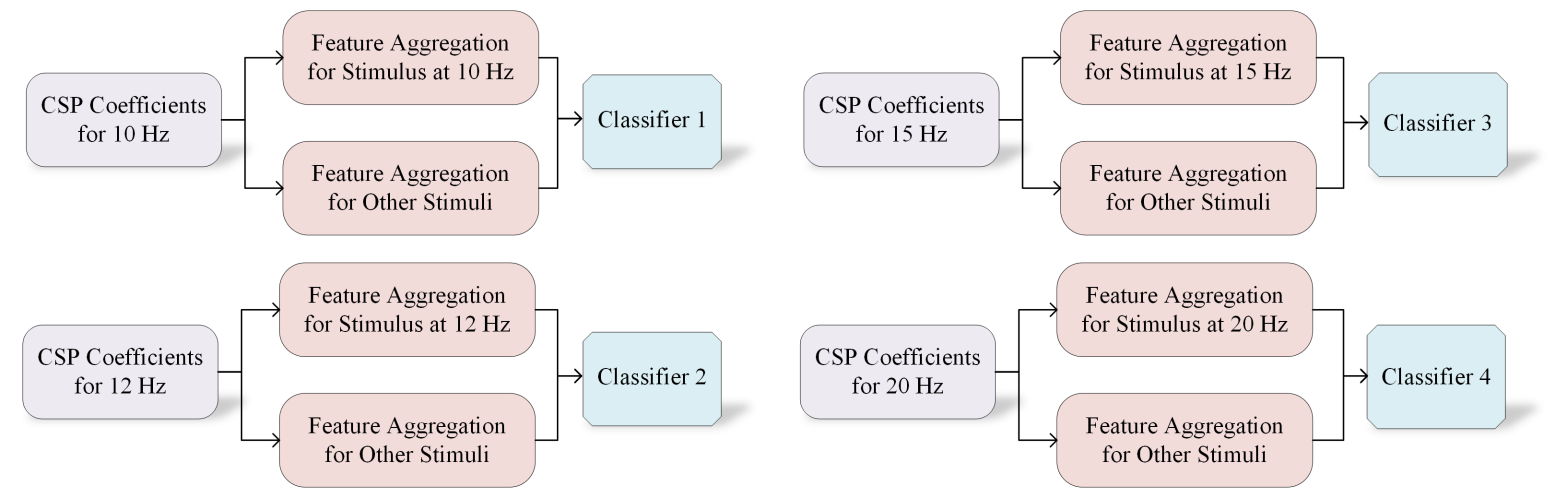
The BCI classification for the four movement stimuli at 10, 12, 15, and 20 Hz using the CSP algorithm.

Figure 9 shows that the CSP coefficients corresponding to the four target frequencies are aggregated and converted into feature vectors for brain activity classification with machine learning. The CSP coefficients for each data segment are calculated for the target and nontarget stimulation frequencies. Specifically, two CSP coefficients are extracted for each frequency, resulting in eight CSP coefficients for each segment of the four target frequencies. Out of the 16 EEG channels, 8 channels were selected for data analysis and classification, as described in Section 4.2. Focusing on these channels ensured that the data contained meaningful information for the classification task while reducing computational complexity. The CSP coefficients were calculated from the signals of these selected channels. The feature vectors were then input into frequency-specific classifiers for brain wave activity classification. The classifiers used in this study included LDA, SVM, and multilayer perceptron (MLP).

The experiment evaluated the effectiveness of the developed SSVEP-BCI system for classifying brain activity. In the preliminary trial, a participant’s EEG data were segmented into 2816 segments, and a feature dataset with a shape of 2816 × 2 was generated for each target frequency. The feature dataset was split in a ratio of 75:25 for training and validation of the classifiers. Stratified sampling was employed to ensure a balanced representation of each target frequency during the split. This approach guaranteed that each movement (i.e., each target frequency) was proportionally represented in both the training and validation sets, thereby preserving the distribution of the target classes across both subsets. Table 1 lists the accuracy of the brain activity classification for the participant in this trial. The data in this table represent the results of only one participant.

**Figure 10:**
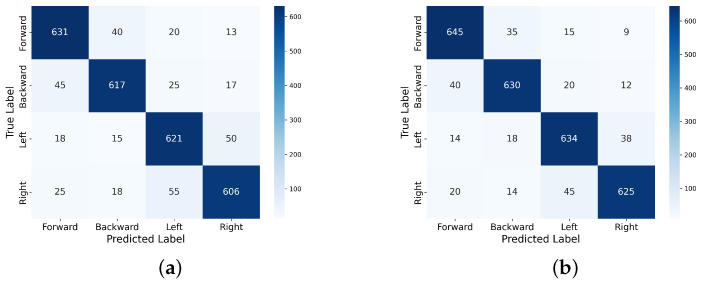

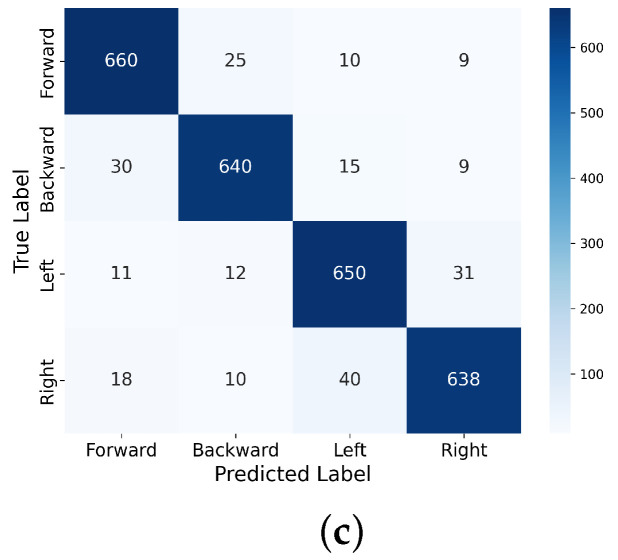
Confusion matrices for the classification of brain activity. (**a**) LDA classifier. (**b**) MLP classifier. (**c**) SVM classifier.

According to the confusion matrices shown in Figure 10, each classifier exhibits different performance characteristics when classifying the four motor movements. Figure 10a shows higher levels of misclassification, particularly between backward and right movements, highlighting the limitations of LDA in distinguishing complex, overlapping EEG patterns. Figure 10b presents higher accuracy results than LDA. However, it still has difficulty distinguishing actions such as left and backward movements. Figure 10c shows the best performance, with a lower overall misclassification rate. Nevertheless, movements such as left and right remain challenging for classifiers in EEG-based systems due to their similar neural signatures. The best classification accuracies for movements of forward, backward, left, and right are 90.72%, 90.83%, 90.33%, and 88.27%, respectively. Overall, an average accuracy of 89.88% can be achieved with the SVM.

### 6.2. Comparison with Similar Methods

Table 2 presents a comparison of the classification accuracy of the proposed system with that of other similar methods in the literature. It shows that the proposed system performed better in terms of classification accuracy in most cases, except in the two cases where complicated feature extraction algorithms and deep neural networks were used. Lim and Ku [35] implemented a multiple-command single-frequency SSVEP-based BCI system using flickering action video. There were three actions, including left, right, and rest, in their experiments. The overall classification accuracy was 74.6% for their SSVEP system. In another similar SSVEP-based BCI system, Asheri et al. [31] used three different frequencies (12, 15, and 20 Hz) of visual stimulation and the harmonics of these frequencies to detect three stimuli. They achieved an average classification accuracy of 89.37% using the CSP and SVM methods. This is slightly lower than the 89.88% accuracy achieved with our system. However, they obtained a much higher average accuracy of 95.45% after using the filter bank CSP (FBCSP) to improve the selection of key temporal–spatial features from EEG signals and widen the target frequency band. This is better than the results achieved in our experiment and could potentially be very useful in improving the performance of our system in the future.

Table 2 also shows that by using a deep neural network (DNN) combining a convolutional neural network (CNN) with a bidirectional long short-term memory (LSTM) network, a classification accuracy of 89.62% was obtained with a dataset collected from a multifunctional robot based on SSVEP-BCI by Ban et al. [57]. This is comparable with the results of our system. Again, using a complex CNN-LSTM network but combined with spectral normalization and label smoothing technologies, Pan et al. [58] achieved a better accuracy of 90.75% with their SSVEP-BCI system. It is noticeable that using DNNs could potentially improve the classification accuracy of SSVEP-BCI systems.

**Table 2:** Comparison of classification accuracy with similar methods reported in the literature.

Method	Frequency (Hz)	Accuracy (%)
Asheri et al. [31]	12, 15, 20	89.37
Asheri et al. (FBCSP) [31]	12, 15, 20	95.45
Lim and Ku [35]	20	74.60
Pan et al. [58]	8, 10, 12, 15	90.75
Ban et al. [57]	8, 10, 12, 15	89.62
Proposed	10, 12, 15, 20	89.88

### 6.3. Limitations

The evaluation presented in this paper is based on a preliminary trial of the developed SSVEP-BCI system with only a limited number of participants, aiming to examine the system’s usability. The dataset is rather small, and its classification accuracy is not high enough to control a device reliably yet. In order for a participant to interact with the environment and control devices reliably, such as maneuvring a virtual vehicle in the developed virtual environment in this work, higher accuracy of classification is needed for the SSVEP-BCI system. Therefore, there is much to be improved in terms of the classification accuracy of this method. It is envisaged that much higher accuracy will be achieved with an enlarged dataset collected from more participants and the utilization of deep machine learning for classification in the future.

We did not evaluate the response time of the SSVEP-BCI system. The SSVEP-BCI system needs to minimize the latency between detecting user intent and the actual action in the environment. A low-latency response is critical for tasks that require quick reactions, such as avoiding obstacles or executing sudden maneuvers in real time. The ITR [59], or effective bit rate, is a suitable metric for evaluating the response time, as it aids in assessing different target identification algorithms by combining the identification speed and accuracy of SSVEP-BCI systems. The ITR of the developed system will be evaluated thoroughly in the next phase of this work.

### 6.4. Future Work

This work aims to investigate the feasibility and effectiveness of using SSVEP-BCIs in human activity monitoring and assessment and to provide an open-source EEG dataset for the SSVEP-BCI community for future research. This paper presents the results of a preliminary trial of the system, which involved a single participant. After the preliminary trial, 20 participants will be formally recruited for system testing and data collection as planned. Appropriate ethical approval was obtained for this work.

The performance of the SSVEP-BCI system will need to be further improved, particularly by raising the classification accuracy for participants maneuvering devices with BCIs. Using the FBCSP and harmonic frequencies of stimuli has been shown effective in obtaining more accurate results [31]. DNNs, such as a combination of CNN and LSTM networks, will also be used to further improve the classification accuracy of EEG signals.

## 7. Conclusions

An SSVEP-BCI system that enables hands-free control of virtual targets in a developed virtual environment was proposed in this paper, allowing participants to control the target using only brain activity. In addition, a publicly available multicategory SSVEP-BCI dataset will be created based on data collected from participants performing forward, backward, left, and right movements in the virtual environment to simulate directional control commands. We conducted preliminary trials with limited participants on the system, and the results showed that a movement classification accuracy of 89.88% was achieved using the ICA and CSP algorithms for feature extraction and conventional machine learning algorithm SVM as classifier. The preliminary trial results demonstrated the feasibility and effectiveness of the proposed SSVEP-BCI system in identifying movement stimuli and showed its potential in human activity monitoring and assessment. In the future, a larger dataset with more participants will be collected, and deep learning models and better feature extraction methods will be employed to improve the classification accuracy significantly.

## Figures and Tables

**Table 1:** Classification results of brain activity with specified target frequencies for four stimulations.

			Accuracy (%)	
**Action**	**Target Frequency (Hz)**	**LDA**	**MLP**	**SVM**
Forward	10	89.68	90.43	90.72
Backward	12	87.59	90.83	90.20
Left	15	88.27	88.67	90.33
Right	20	86.06	87.82	88.27
Average		87.90	89.43	89.88

## Data Availability

The datasets presented in this article are not readily available because the data are part of an ongoing study.
